# Do medical student attitudes towards patients with chronic low back pain improve during training? a cross-sectional study

**DOI:** 10.1186/1472-6920-12-10

**Published:** 2012-03-19

**Authors:** Hayley Morris, Cormac Ryan, Douglas Lauchlan, Max Field

**Affiliations:** 1 College of Medical, Veterinary and Life Sciences, University of Glasgow, Glasgow, UK; 2 School of Health and Social Care, Teesside University, Middlesbrough, UK; 3 School of Health and Social Care, Glasgow Caledonian University, Glasgow, UK

**Keywords:** Back pain, HC-PAIRS, Attitudes, Evidence based medicine

## Abstract

**Background:**

Health care professionals with positive attitudes towards the functional abilities of patients with low back pain are more likely to encourage activity and avoidance of rest as per recommended guidelines. This study investigated whether medical student training fosters positive attitudes towards patients with back pain and their ability to function.

**Methods:**

First (n = 202) and final (n = 146) year medical students at the University of Glasgow completed the Health Care Professionals' Pain and Impairment Relationship Scale (HC-PAIRS) questionnaire. This measures attitudes of clinicians towards the functional ability of patients with back pain. A group of first (n = 62) and final year (n = 61) business students acted as non-health care controls. Attitudes were compared using two-way ANOVA with year of study and discipline of degree as independent variables.

**Results:**

Both year of study [F(1,465) = 39.5, p < 0.01] and discipline of degree [F(1,465) = 43.6, p < 0.01] had significant effects on total HC-PAIRS scores and there was a significant interaction effect [F(1,465) = 9.5, p < 0.01]. Medical students commenced their course with more positive attitudes than non-health care students (65.7 vs. 69.2 respectively; p < 0.01) - lower scores translating into more positive attitudes. In their final year, the difference between the two student groups had widened (56.4 vs. 65.3; p < 0.01).

**Conclusions:**

Undergraduate medical training promotes positive attitudes towards the functional ability of patients with back pain, suggesting that students may be more likely to develop an evidence-based approach to this patient group after qualification. Some adjustments to training may be warranted to encourage a more positive shift in attitudes.

## Background

Low back pain (LBP) affects > 80% of the population [1], with recurrent symptoms resulting in absence from work in 37% of patients [2]. The estimated total cost of LBP to the United Kingdom is 1-2% of the gross domestic product [3], a drain attributable to direct health care costs and lost productivity. Current LBP guidelines advocate continued activity and early return to work [2,4], and the interventions advocated in these guidelines facilitate better patient outcomes and cost effective practice [5,6]. However, compliance of medical professionals with these treatments tends to be poor [7] and seems not to have changed in recent years [8,9].

Health care providers with negative attitudes to chronic LBP (CLBP) are more likely to recommend advice contrary to current guidelines including prolonged absence from work. However, those with more positive attitudes are more likely to follow evidence based guidelines [9-11]. The Health Care Providers' Pain and Impairment Relationship Scale (HC-PAIRS) questionnaire assesses the attitudes, beliefs and expectations of health care professionals regarding CLBP and the patient's ability to function [12,13]. Using the HC-PAIRS questionnaire, previous studies have shown that inclusion of back pain specific modules during training has a positive influence on the attitudes of physiotherapy students [14].

It is important that current medical graduates develop the correct attitudes to CLBP patients during training. This study used the HC-PAIRS questionnaire to investigate whether attitudes to CLBP patients alter during undergraduate training, to ascertain whether these students are more likely to use best practice as future practitioners.

## Methods

### Participants and recruitment

First and final year University of Glasgow medical students were recruited during weekly lectures, with all students in attendance invited to participate. Business studies students at Glasgow Caledonian University acted as non-health care controls. The control group data has been previously published elsewhere [15]. Basic demographic characteristics were recorded, as was history of previous and current LBP. Participants were excluded if they had previously undertaken health care degrees.

### Outcome measures

The primary outcome measure used was the total score from the HC-PAIRS questionnaire [12]. It consists of 15 items on a 7-point Likert scale. Scores range from 15 to 105, with lower scores indicating more positive attitudes. Secondary outcomes were the individual subscales within the HC-PAIRS; functional expectations [9 to 63], social expectations [4 to 28], need for a cure [3 to 21], and projected cognition [2 to 14].

### Musculoskeletal system and training in back pain management

At the University of Glasgow, all medical students take part in standard training covering the musculoskeletal system, commencing in year 1 when students learn about basic anatomy and physiology of joints and the range of joint movements. In year 2, students study Problem Based Learning (PBL) scenarios based on acute knee pain, back pain, muscle disease and fracture management. PBL sessions are supported by fixed resource sessions, plenary lectures and clinical skills sessions based on the Gait Arms Legs and Spine (GALS) examination [16]. In year 3, students gain further experience studying rheumatoid arthritis and osteoarthritis and bone pain with osteomalacia using similar techniques. This forms the basis for student learning prior to exposure to patients with musculoskeletal disease including those with back pain during hospital and General Practitioner attachments in the final two years of the course [17].

Non-health care students at Glasgow Caledonian University received no formal training on back pain management during their four year degree course

### Ethics and consent

Ethical approval was obtained from the West of Scotland Research Ethics Committee and the Glasgow Caledonian University School of Health Research and Ethics Committee. All participants provided written informed consent, in accordance with the Declaration of Helsinki.

### Data analysis

Data was analysed using SPSS version 18.0. All data was initially analysed for normal distribution and subsequently using two way ANOVA and post hoc analysis was undertaken using t-tests. A p-value of < 0.05 was considered statistically significant.

## Results

### Participants

A total of 202/240 (84%) and 146/243 (64%) first and final year medical students completed the questionnaires with a female:male ratio of 0.96:1 and 1.86:1 respectively. Five participants were excluded as they had qualified with a previous health care degree. Nineteen incomplete questionnaires were not analysed.

A total of 62/100 (62%) first and 61/94 (64%) final year business studies students completed the questionnaire fully (female:male ratios of 2.2:1 and 6.6:1). None had previous health care degrees. The non-health care student group were younger (20 ± 3 vs. 21 ± 3, p < 0.01) with a greater percentage of females (78% vs. 59%, p < 0.01). There was no statistical difference between the two groups for previous (43% vs. 38%, p = 0.28) or current low back pain prevalence (7% vs. 8%, p = 0.64).

### Analysis of HC-PAIRS questionnaires

Results of HC-PAIRS total scores were normally distributed so primary analysis was undertaken using a two-way ANOVA with year of study and discipline of degree as independent variables and age as a covariate. Both year of study [F(1,465) = 39.5, p < 0.01] and discipline of degree [F(1,465) = 43.6, p < 0.01] had a significant effect on total HC-PAIRS scores and there was a significant interaction effect [F(1,465) = 9.5, p < 0.01].

**Figure 1:**
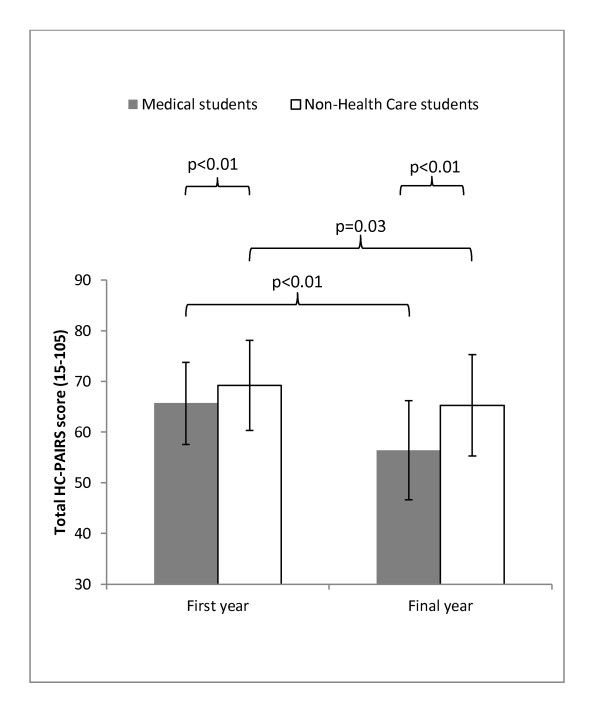
**Attitudes in first and final year students: comparison of total HC-PAIRS scores**. Shows the total HC-PAIRS scores in medical students and those on a non-health care course with lower scores equating with more positive attitudes. Medical students start their course with better attitudes. The improvement in attitudes during training is seen in both groups but those in medical students show the greater change. *Parametric p-values were calculated using independent t-tests.

Figure 1 shows a comparison between total HC-PAIRS scores for medical and non-health care students. Lower scores relate to more positive attitudes in HC-PAIRS questionnaires. Medical students started their course with more positive attitudes than non-health care students (mean difference -3.4; 95% CI -5.8 to -1.1; p = < 0.01). Within groups from first to final year, medical students had developed even more positive attitudes with a mean difference of -9.2 (95%CI -11.1 to -7.3; p = < 0.01) as compared to -3.9 points (95%CI -7.2 to --0.5; p = < 0.03) for non-health care students. In their final years, the difference between the medical and non-health care student groups had widened to a mean difference of -8.9 (95%CI -11.9 to -6.0; p < 0.01).

**Figure 2:**
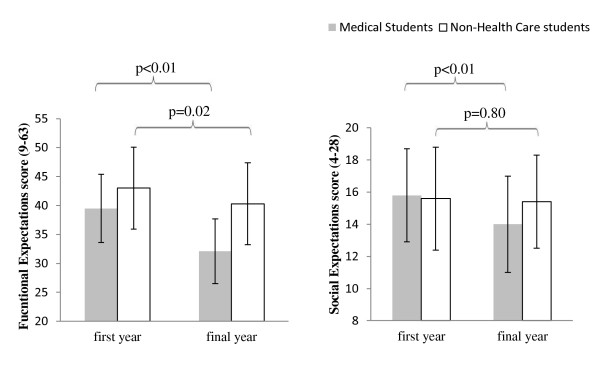
**Functional and Social Expectation subscales scores in first and final year students**. Shows the Functional Expectations (left panel) and Social Expectations (right panel) score for the medical and Non-Health care students. Functional Expectations improved by a statistically significant amount for both student groups from first to final year. Only the Medical student group significantly improved for Social Expectations from first to final year.

Figure 2 shows results of post-hoc comparisons between first and final year students. In addition to the improvement in total HC-PAIRS scores, analysis of the scores for functional expectations also improved for both student groups during training. However, this score enhancement was again greater for medical students (mean difference - 7 5; 95% CI -8.8 to -6.1; p = > 0.01) than for non-health care students (mean difference - 2.7; 95%CI -5.0 to -0.4; p = < 0.02). By comparison social expectations scores only improved for the medical students (mean difference -1.8; 95%CI -2.4 to -1.2; p = < 0.01), and not for non-health care students (-0.1; 95%CI -1.2 to 1.0; p = 0.80). Neither group showed an improvement on the need for a cure or projected cognitions subscales (data not shown).

Only the Medical student group significantly improved for Social Expectations from first to final year. There was no significant difference between females and males for total HC-PAIRS score for medical (62.4 ± 8.7 vs. 61.0 ± 11.5; p = 0.19) or non-health care students (67.3 ± 9.1 vs. 67.2 ± 11.5; p = 0.96). Similarly, the effect of previous low back pain and current low back pain was assessed for the 348 medical students to address whether personal experience could affect attitudes. No significant difference in total HC-PAIRS scores were seen between those with a history of previous low back pain and those without (60.9 ± 10.3 vs. 62.4 ± 9.8, p = 0.17). Nor was there a significant difference between those with and without a current history of low back pain (62.9 ± 10.6 vs. 61.8 ± 9.9, p = 0.59).

## Discussion

This is the first study to address medical student attitudes towards patients with CLBP. The results show that after undergraduate training medical students develop more positive attitudes towards CLBP patients and their ability to function. Breakdown of the scores into different HC-PAIRS subscales shows that medical students undergo an improvement in both functional (patients' ability to undertake activities of normal daily living) and social (patients' ability to work and take on family responsibilities) expectations when considering patients with CLBP. Hence undergraduate medical students do develop the appropriate attitudes towards CLBP during training and this reaches equivalent levels to those attained by final year UK physiotherapy students [15]. Extrapolating from the evidence for correlation of attitudes and management of CLBP patients by qualified medical practitioners, these results imply that medical students should be more likely to use an evidence-based approach with these patients after graduation. However, final year medical student scores are not as positive as values recorded for a cross section of practicing USA community health care providers [10]. This indicates that there remains an important role for postgraduate medical education about CLBP in the United Kingdom. It would be interesting to follow these students during their postgraduate training to assess if attitudes continue to change and if this is indeed reflected in their practice.

Comparisons between the two student groups at the start of their courses shows that medical students have more positive attitudes to CLBP than controls. The reason for this difference remains to be established but may result from potential medical students having more positive attitudes and so "self-selecting" to enter medical degree courses. Equally, it could be that students with positive attitudes to illnesses in general are more likely to be successful in their application and interview prior to joining the University.

Interestingly, both student groups improved their attitudes to CLBP during their courses. The explanation for the small improvement in the total HC-PAIRS and functional expectations scores in the business studies students remains unclear as they receive no formal training about CLBP. Student age was not a significant covariant in the analysis and hence would be insufficient to explain this phenomenon but increased maturity and awareness of CLBP which could exert a small influence on both student groups might be a cause. Nevertheless, the alterations in final year medical student scores were larger and the unique improvement in social expectations subscale scores confirms that positive attitudes can be reinforced during appropriate training.

Scores on the projected cognition subscale (i.e. patients' ability to concentrate despite having low back pain) and the need for a cure subscale (i.e. patients' need for a cure before they can function well) were no different between first and final year medical students (data not shown). This indicates that training has no effect on medical student perceptions of CLBP patients' abilities in these two areas. It could be argued that if the present holistically based training module on CLBP addressed these details then these two parameters should also change. In previous studies of physiotherapy students these parameters were also unchanged after training [15], raising some uncertainty about the validity of these two subscales of the HC-PAIRS questionnaire [10]. Nevertheless, in order to be sure that there are no areas being neglected during student training about CLBP, these need to be reviewed to ensure adequate coverage.

The HC-PAIRS questionnaire has been shown to be one of the most valid and reliable measures with which to assess attitudes to CLBP [13] and hence was used in this study. One limitation was the cross-sectional nature of the study comparing separate groups of first and final year students. Analysis of one medical student group over five years would establish whether changes occur gradually or subsequent to modules where back pain is studied in detail. Certainly, physiotherapy student attitudes improved after introduction of a specific back pain module into their training programmes [16] suggesting that specific training can have a positive effect. Longitudinal studies would also allow investigation of the influence of clinical placements on student attitudes to CLBP. A second limitation is that this study only addresses responses in a single centre and results may not generalise to all medical schools. Nevertheless, these results do imply that the HC-PAIRS questionnaire can be successfully applied, and can therefore be used to objectively assess the impact of undergraduate medical training about CLBP.

Another limitation of the study is the poorer recruitment rate in final year (64%) compared to first year (84%) medical students. All students were invited to participate in the study at a weekly lecture during the first semester of the year. Hence, final year students were recruited a few weeks before their final written examinations and the poor response rate follows a general trend for poorer attendance at lectures around this time. It is therefore conceivable that some of the apparent improvement in scores is through self-selection - that those more likely to maintain good attendance at lectures are more likely to have more positive attitudes. Again this is an issue we hope to resolve with the paired data that will accrue during the annual follow-up of the students in this first year cohort.

A number of medical students reported experiencing current or previous low back pain. It might have been predicted that this could influence student responses. The results from medical students suggest that it is training which shapes the student attitudes rather than their personal experiences. This is in keeping with a similar assessment of physiotherapy students who similarly seemed to have the ability to remain objective about their own LBP when completing the HC-PAIRS questionnaire [18].

It is important to ensure that medical practitioners have the appropriate attitudes about CLBP if patients are to be given appropriate treatment to reach their full potential. If present clinicians have poor compliance with guidelines for CLBP management [8,9] then there is clearly a need for improved postgraduate training. It is recognised that clinicians with more positive attitudes are more likely to comply with CLBP treatment guidelines [10]. Hence the fact that medical students graduate with positive attitudes implies that new generations of clinicians will enter postgraduate training with a background that should lead to evidence based practice with CLBP patients and in turn to improved patient outcomes [19].

## Conclusions

Medical training promotes positive attitudes towards patients with back pain and their functional abilities in comparison to a non-healthcare education. A positive shift in attitudes towards the ability of patients to perform activities of daily living and perform social roles such as work and family responsibilities were identified. The magnitude of improvement was similar to that reported for other healthcare students groups. Based on evidence from studies of qualified medical practitioners, the development of positive attitudes in students may increase the likelihood that following graduation they will follow clinical guidelines and become evidence-based practitioners with their back pain patients. There were some specific attitudes, such as the ability of patients to function cognitively despite their pain which did not improve during the five year programme and adjustments to training may be warranted to encourage a more positive shift in these attitudes.

## Competing interests

The authors declare that they have no competing interests.

## Authors' contributions

MF and CR conceived of the study, participated in its design, collected data and helped to draft the manuscript. CR performed the statistical analysis. HM was involved in processing the HC-PAIRS questionnaires and drafting the manuscript. DL was involved in the study design development and helped to draft the manuscript. All authors reviewed and approved the final manuscript.

## Authors' information

No additional information.

## Pre-publication history

The pre-publication history for this paper can be accessed here:

http://www.biomedcentral.com/1472-6920/12/10/prepub
